# Gene‐diet quality interactions on haemoglobin A1c and type 2 diabetes risk: The Airwave Health Monitoring Study

**DOI:** 10.1002/edm2.74

**Published:** 2019-07-11

**Authors:** Rebeca Eriksen, Rachel Gibson, Maria Aresu, Andy Heard, Queenie Chan, Evangelos Evangelou, He Gao, Paul Elliott, Gary Frost

**Affiliations:** ^1^ Nutrition and Dietetic Research Group, Faculty of Medicine Imperial College London UK; ^2^ Department of Nutritional Sciences, School of Life Course Sciences King's College London London UK; ^3^ Department of Epidemiology and Biostatistics, MRC‐PHE Centre for Environment and Health, School of Public Health Imperial College London UK

**Keywords:** diet, genetic risk, type 2 diabetes

## Abstract

**Introduction:**

Type 2 diabetes (T2D) is multifactorial involving lifestyle, environmental and genetic risk factors. This study aims to investigate the impact of genetic interactions with alcohol and diet quality on glycated haemoglobin A1c (HbA1c) independent of obesity, in a British population.

**Methods:**

Cross‐sectional study of 14 089 white British participants from Airwave Health Monitoring Study and a subsample of 3733 participants with dietary data. A T2D genetic risk score (GRS) was constructed, and its interactions with diet on HbA1c were assessed.

**Results:**

GRS was associated with a higher HbA1c% (*β* = 0.03, *P* < 0.0001) and a higher risk of prediabetes (OR = 1.09, *P* < 0.0001) and T2D (OR = 1.14, *P* = 0.006). The genetic effect on HbA1c% was significantly higher in obese participants (*β* = 1.88, *P*_interaction_ = 0.03). A high intake of wholegrain attenuated the effect on HbA1c% in high‐risk individuals *P*_interaction_ = 0.04.

**Conclusion:**

The genetic effect on HbA1c was almost doubled in obese individuals, compared with those with a healthy weight, and independent of weight, there was a modest offset on HbA1c in high‐genetic‐risk individuals consuming a diet high in wholegrain. This supports the importance of a healthy diet high in wholegrains and along with maintaining a healthy weight in controlling HbA1c among high‐genetic‐risk groups.

## 1 INTRODUCTION

Worldwide over 382 million people are estimated to have type 2 diabetes (T2D).^1^ People with T2D have a fivefold risk in developing cardiovascular disease (CVD) and are 1.6 times more likely to die prematurely, compared with those without T2D.^2^ Early intervention at the critical stage, prediabetes may halt the development of T2D and further reduce the risk of CVD and related premature mortality worldwide.^1^

The aetiology of T2D is multifactorial, with obesity, poor diet quality, physical inactivity and genetic factors identified as key risk factors.^2^ Genome‐wide association studies (GWAS) have identified over 80 T2D‐associated loci 3, 4 of which 70 loci are associated with glycaemic trait^5^, ^6^; most of these are not associated with obesity markers. Single nucleotide polymorphisms (SNP) have small‐to‐moderate effects and account for only a small proportion of the heritability of T2D.^3^, ^7^, ^8^ To increase the predictability, SNPs are combined into genetic risk scores.

Lifestyle factors such as diet, alcohol and weight are modifiable key components in the development of T2D.^2^ Although response to diet is variable, emerging evidence suggests that these lifestyle factors may modify the genetic effect on development of T2D.^9^, ^10^, ^11^ Dayeh et al^10^ showed epigenetic changes in over 853 T2D‐related genes (1649 CpG sites) associated with age and environmental factors (physical activity, adiposity), resulting in modifications to various glycaemic traits. The US National Health and Nutrition Examination Surveys found that the genetic effect on T2D outcome was modified (reduced) in individuals with a diet high intake of carbohydrates and fibre.^12^ Most interaction dietary studies to date have only looked at genetic effects and single nutrients derived from food frequency questionnaires.^13^ EPIC‐InterAct highlighted the importance of studying the combined effect of these SNPs as a genetic risk scores (GRS) for T2D yet; no interactions were found with any single nutrients.^14^ However, evidence suggests modification of diabetes risk factors is related to a profile of diet quality rather single nutrients.^15^ There is a need for a more comprehensive assessment of high‐quality diet data, dietary profiling with genetic interactions on risk of T2D, to fully understand the complexity of dietary intake and the combined effect of these SNPs as genetic a genetic risk score.

The aim of the study is to investigate associations between the combined effect of genetic variants and HbA1c among a white British population group, and if these associations are modified by interactions with lifestyle factors including diet quality (dietary profiling) and alcohol independent of obesity markers; body mass index (BMI) and waist circumference.

## 2 METHODS

### 2.1 Study design and participants

Members of the police forces in Great Britain were eligible for inclusion in the Airwave Health Monitoring Study. Further details of the recruitment procedures and data collection methods have been described elsewhere.^16^ The present study comprises a random sample of 14 089 white British participants who took part in a health screen, genetic testing and a subsample of 3733 participants who also provided dietary data between 2007 and 2012. All biological sampling and dietary data were collected in the same period during participants first visit to the clinic.

### 2.2 Ethics approval

Participants provided written informed consent, and the study had ethics approval from the National Health Service Multi‐Site Research Ethics Committee (MREC/13/NW/0588).

### 2.3 Data collection

The health screenings were carried out in dedicated Airwave Health Monitoring Study clinics using a standard protocol. Trained nurses conducted all clinical examinations.

#### 2.3.1 Anthropometry

Height and sitting height were measured using a Marsden H226 portable stadiometer and weight using a Marsden digital weighing scale. Waist circumference was measured using a Wessex‐finger/joint measure tape (Seca 201; Seca). For all anthropometrics, two measurements were taken and the average was used.

#### 2.3.2 Blood samples

All samples were taken nonfasting. Tests were performed using serum samples except HbA1c, which was performed using whole EDTA blood sample and glucose determination, which was performed using fluoride/oxalate sample tube. Samples were measured using an IL 650 analyser (Instrumentation Laboratory).

#### 2.3.3 DNA extraction and genotyping

Participants were genotyped using the Illumina HumanCore Exome‐12v1‐1 BeadChip array and imputed using the 1000 Genomes phase 3 reference panel in MiniMac. For this analysis, genotypes for the SNPs of interest were assigned based on the highest genotype probability if the SNP was imputed. Additional quality control filters were applied to the included SNPs in plink version 1.07^17^; 282 participants and one SNP were excluded based on Hardy‐Weinberg equilibrium (*P* < 0.001), minor allele frequencies (MAF) (<0.01), SNP missingness and individual missingness rates (<0.01). Detailed quality control protocol is described in Supporting Information.

#### 2.3.4 Socio‐demographic

Health and lifestyle data were collected via a self‐administrated electronic questionnaire, which the participant completed during their clinic visit. Variables used for this study included age, gender, ethnicity, smoking status, diagnosed diseases, medication usage and physical activity, estimated using the International Physical Activity Questionnaire short version. The metabolic equivalent of task (MET) in minutes per week was calculated for each participant and categorized as high (at least 60 minutes/d of at least moderate‐intensity activity), medium (at least 30 minutes/d of at least moderate‐intensity activity) and low (no activity or less then medium category).^18^

### 2.4 Dietary data

Dietary intake was assessed using a validated 7‐day food diary (estimated weight).^19^ Recorded dietary intakes were translated into nutrient intake data using dietplan software (Forestfield Software Ltd) which is based on the McCance and Widdowson's 6th Edition Composition of Foods UK Nutritional Dataset (UKN). All dietary coders were trained by a research dietician/nutritionist using a study‐specific operational manual protocol. Detailed description of the coding and diet analysis is reported elsewhere.^20^

Overall quality of dietary intake was determined by the UK Dietary Reference Value index (DRV). The DRV index is an a priori diet quality index that reflects adherence to the UK dietary reference values and guidelines^21^ and has previously been shown to be negatively associated with HbA1c, waist circumference and T2D.^22^ The 16‐point DRV index consists of eight nutritional components: total fat, saturated fat, sodium, fruit/vegetables, fibre, added sugar, total carbohydrates, fish (including oily fish).^22^ In short, mean daily intake of each of the eight components is assessed and scored according to the UK DRV. The sum of the total points provides an overall score between 0 and 16 points; a higher score indicates a more favourable diet.

### 2.5 SNP selection and genetic risk score

The analysis included 87 common SNPs (Table S2) associated with T2D and HbA1c in European‐descent populations extracted from 164 SNPs identified in a systematic literature review in databases, DIAbetes Genetics Replication And Meta‐analysis consortium (DIAGRAM)^3^ and Meta‐Analyses of Glucose and Insulin‐related traits Consortium (MAGIC),^4^, ^6^ and from the National Human Genome Institute Research (NHGIR) GWAS catalogue.^23^ Systematic literature review is detailed in Supplementary Material. SNPs in high linkage disequilibrium (*r* ^2^ < 0.8) were excluded. SNPs located in obesity gene FTO (rs9939609, rs8050136, rs11642841, rs8090011) were excluded given the well‐documented association with obesity which mediates the effect on T2D risk. Diet interaction analyses were conducted for GRS including the SNPs in FTO gene to investigate the diet role when controlling for FTO SNPs. An unweighted poly‐genetic risk score (GRS) was constructed in PLINK for each participant by summing the number of risk alleles present (0 for noncarrier, 1 for heterozygous and 2 for homozygous). The GRS was standardized against the sample population's standard deviation (SD) for appropriate scaling for clinical interpretation: Standardized (centred) GRS = GRS/GRS SD.

### 2.6 Clinical definitions

Type 2 diabetes was defined as HbA1c ≥6.5%, diagnosed T2D or on glucose‐controlling medication.^24^ Prediabetes was defined as HbA1c between 5.7% and 6.4%.^25^ BMI was calculated as weight in kg divided by the square of height in metres. BMI categories were underweight (BMI <18.5 kg/m^2^), healthy (18.5‐24.9 kg/m^2^), overweight (25‐29.9 kg/m^2^) and obese (≥30 kg/m^2^).

### 2.7 Statistical analysis

Baseline socio‐demographic and lifestyle characteristics of participants were compared across GRS tertiles (T) using linear regression for continuous variables and chi‐square test for categorical variables.

Association between the GRS and HbA1c was analysed via multivariable linear models. Both HbA1c and GRS followed a normal statistical distribution. Models were adjusted for covariates including age, gender, BMI, smoking, physical activity, diabetes diagnosis and treatment, based on previous studies.^3^, ^4^, ^5^, ^6^ Associations with prevalence of T2D and prediabetes outcome was analysed via logistic regression models adjusted for above covariates except for diabetes diagnosis and treatment.

Gene‐environment interactions between GRS tertiles and dietary components on HbA1c were analysed via separate multivariable linear models (proc GLM) including one interaction term per model: HbA1c = GRS + interaction variable + (GRS * interaction variable) + covariates. The interaction parameter estimates (beta‐coefficients), standard errors, 95% CI and *P* for interaction were reported. Models were adjusted for appropriate covariates, age, gender, BMI, alcohol, smoking, physical activity, mean energy intake, diabetes diagnosis and treatment, based on previous studies.^9^, ^10^, ^11^ sas version 9.4 (SAS Institute Inc VX) was used for all analysis. Statistical significance threshold was set at *P* < 0.05.

## 3 RESULTS

Descriptive statistics of the sample (n = 14 089) are shown in Table S1. The GRS was not associated with adiposity markers (BMI or waist circumference), socio‐demographic or lifestyle characteristics. Differences across GRS were seen for prevalence of prediabetes and T2D.

In multivariate analyses, GRS was associated with HbA1c. The estimated coefficient in HbA1c was 0.03 (95% CI 0.02, 0.04) per 1 (SD) increase in the GRS (*P* < 0.0001) adjusted for age, sex, BMI, smoking, physical activity, diabetes diagnosis and treatment (Table 1). The GRS was also associated with prevalence of prediabetes and T2D. The OR for having prediabetes increased by 9% (95% CI 1.05‐1.13) and T2D by 14% (95% CI 1.04‐1.24) per 1 (SD) increase in the GRS (Table 2).

**Table 1:** Genetic score association with HbA1c% in the Airwave Health Monitoring Study (n = 14 085)

	Model 1^a^			Model 2^b^			Model 3^c^		
	*β*	95% CI	*P*‐value	*β*	95% CI	*P*‐value	*β*	95% CI	*P*‐value
HbA1c (%)	0.03	0.03, 0.05	<0.0001	0.03	0.03, 0.05	<0.0001	0.03	0.02, 0.04	<0.0001

Abbreviations: CI, confident interval; *β*, beta‐coefficient.

a Adjusted for body mass index, age and sex.

b Adjusted for waist circumference, age and sex.

c Adjusted for age, sex, body mass index, smoking, physical activity, diabetes diagnosis and glucose lowering medication.

**Table 2:** Risk of prediabetes and type 2 diabetes by per one point (standardized) increase in genetic score, the Airwave Health Monitoring Study (n = 14 085)

	Cases/Subcohort	OR^a^	95% CI	*P*‐value
Prediabetes^b^	5253/8820	1.09	1.05, 1.13	<0.0001
Type 2 diabetes^c^	516/13 569	1.14	1.04, 1.24	0.006

Abbreviations: 95% CI, 95% confidence interval; OR, odds ratio.

a Adjusted for age, sex, BMI, smoking, physical activity.

b HbA1c > 5.7 ≤ 6.5% and not on glucose lowering medication or previously diagnosed with diabetes.

c HbA1c ≥6.5% and/or diagnosed with diabetes and/or glucose‐lowering medication.

We observed significant interactions between GRS and intake of wholegrains *P* for interaction 0.04 (Table 3). The effect of the diet interactions on HbA1c was greater in high‐genetic‐risk individuals (GRS T2; *β* = −0.1% per 100 g, 95% CI −0.1, 0.01, GRS T3; *β* = −0.1% per 100 g, 95% CI −0.1, 0.01). No significant interactions were observed for the other dietary variables. All models were adjusted for age, sex, BMI, smoking, alcohol, energy intake, physical activity, diabetes diagnosis and treatment. Sensitivity analyses showed that these diet effect modifications on HbA1c were similar to a GRS that also included SNPs in FTO and after controlling for obesity markers (Table S4).

**Table 3:** The effect of genetic‐diet interactions on HbA1c across genetic risk tertiles, the Airwave Health Monitoring Study (n = 3733)

Dietary components	GRS tertile 1	GRS tertile 2		GRS tertile 3		
	Lowest risk	n = 1485		Highest risk		
	n = 1085			n = 1161		
		*β* a	95% CI	*β* a	95% CI	*P*_interaction_
DRV score^b^	Ref	−0.01	−0.02, 0.003	−0.01	−0.03, 0.01	0.15
Carbohydrates (per 10 g)	Ref	−0.001	−0.01, 0.005	−0.04	−0.01, 0.002	0.41
Fibre (per 10 g)	Ref	−0.02	−0.1, 0.1	−0.04	−0.1, −0.004	0.71
Fruit, vegetable (per 100 g)	Ref	−0.02	−0.04, −0.01	−0.01	−0.03, 0.01	0.12
Wholegrains (per 100 g)	Ref	−0.1	−0.1, 0.01	−0.07	−0.1, 0.01	0.04
Total fat (per 10 g)	Ref	0.005	−0.01, 0.02	0.01	−0.01, 0.03	0.64
Saturated fat (per 10 g)	Ref	0.01	−0.03, 0.05	0.006	−0.03, 0.05	0.86
Added sugars (per 10 g)	Ref	−0.002	−0.01, 0.009	−0.006	−0.02, 0.01	0.55

Abbreviations: CI, confident interval; DRV, dietary reference value score; GRS, genetic risk score; *P* interaction; *P*‐value type III error; *β*, beta‐coefficient.

a Estimated effect on HbA1c% per increase in nutrient variable interaction with GRS tertiles adjusted for age, gender, smoking, alcohol, energy intake, physical activity, BMI, diabetes diagnosis and treatment.

b Coefficients represent per 1 point increase in DRV score.

There was a significant interaction between GRS and across BMI categories. We observed a greater effect of GRS on HbA1c among obese participants (BMI <30 kg/m^2^), *β* = 1.88% (95% CI 0.41, 3.34), *P* for interaction 0.03. No interaction was observed for across alcohol consumption group (Table 4).

**Table 4:** The effect of genetic interactions on HbA1c, across categories of alcohol consumption and body mass index, the Airwave Health Monitoring Study (n = 3733)

Interacting variables	n	HbA1c (%)		*P*_interaction_
		*β* a	95% CI	
Alcohol categories				
No alcohol intake	767	Ref		0.6
Within UK allowance^b^	1670	−0.19	−1.57, 1.18	
Above UK allowance^c^	1296	0.34	−1.08, 1.76	
BMI categories				
BMI (18.50‐24.99 kg/m^2^)	1273	Ref		0.03
BMI (25‐29.99 kg/m^2^)	1741	0.11	−0.01, 1.22	
BMI (>30 kg/m^2^)	711	1.88	0.41, 3.34	

Abbreviations: Beta, beta‐coefficient interaction effect; BMI, body mass index; CI, confident interval; HbA1c, glycated haemoglobin.

a Beta‐coefficient for the estimated difference in HbA1c% per unit increase in genetic risk score interacting with alcohol or BMI category adjusted for age, gender, smoking, physical activity, (BMI), (alcohol). Note: alcohol included in BMI model and BMI in alcohol model.

b >0 unit and <2 units of alcohol/d.

c >2 units of alcohol/d.

## 4 DISCUSSION

In this large, well‐characterized occupational cohort, we found, among individuals of European ancestry at genetically high risk of diabetes, that consuming a high‐quality diet and high wholegrain intake was associated with a beneficial modification of the genetic risk. This could have important future public health application in identifying genetically high‐risk individuals before the onset of disease, as early intervention may help slow or halt the development of T2D and CVD.^26^

We found attenuation of the genetic effect on HbA1c among participants consuming a diet high in wholegrains, aligning with WHO's dietary guidelines for prevention and management of T2D.^2^ The diet interactions were greater in higher‐risk individuals (persons in the two highest tertiles for GRS), suggesting that such individuals may benefit more from maintaining a healthy diet. Similar findings were reported in individuals of European ancestry.^2^, ^27^, ^28^, ^29^, ^30^, ^31^ The Prevención con Dieta Mediterránea study (PREDIMED) analysing 4.8‐year follow‐up data (n = 7018). They observed interactions between a higher diet quality (Mediterranean diet) and rs7903146 SNP (TCF7L2 gene) in relation to fasting glucose,^27^ supporting the suggestion that individuals of European ancestry at high genetic risk of diabetes consuming a high‐quality diet could beneficially modify their disease risk.

Interactions between wholegrains and single genetic variants on glycaemic trait and T2D risk have previously been reported. The European Prospective Investigation of Cancer (EPIC) study reported that an interaction with wholegrain intake inversely modified the association between rs7903146 (*TCF7L2* gene) and T2D risk.^31^ The Malmö Diet and Cancer Study (n = 24 799) showed interactions between intake of dietary fibre and the same SNP rs7903146 (*TCF7L2*) associated with 32% lower T2D incidence among participants with the highest fibre intake (OR: 1.24, 95% CI 1.04‐1.47) compared to those with lowest fibre intake (OR: 1.56, 95% CI 1.31‐1.86, *P*_interaction_ = 0.05).^30^ The same study reported a reduced effect of SNP rs10423928 (*GIPR* gene) on T2D incidence in individuals who had the highest intake of carbohydrate (OR = 1.23, 95% CI: 5%‐39%) compared to those with the lowest carbohydrate intake (OR = 1.69, 95% CI: 29%‐86%). Our study did not observe any interaction with neither fibre nor carbohydrates despite these variables being highly correlated with wholegrains. However, the study sample size (n = 3733) was significantly smaller sample size than the Malmö Diet and Cancer Study (n = 24 799), which can affect power in such interaction studies.

The modest protective effect of healthier diets on HbA1c reported here, although not clinically important at an individual level, is valuable at a public health level, where a small change in the distribution of HbA1c would result in a population reduction in future T2D. The UK Prospective Diabetes study (n = 3642) observed that a 1% reduction in HbA1c was associated with a reduction in relative risk of T2D of 21% (95% CI 12%‐24%), number of CVD complications (37%, 95% CI 33%‐41%) and mortality from diabetes (21%, 95% CI 15%‐27%).^32^

Finally, interactions were seen among obese individuals (BMI ≥30 kg/m^2^) with nearly twofold increase in HbA1c%, which is in line with previously reported findings.^7^, ^33^ The Prevención con Dieta Mediterranea RCT (n = 7018) also reported significant interactions among obese participants.^33^

### 4.1 Biological plausibility

This cross‐sectional study cannot delineate the mechanisms underlying the observed interactions. Several lines of evidence suggest that such interactions between these T2D‐associated genetic variants and nutrient groups are plausible. The beneficial bioactivities of a healthy diet such as balancing energy intake and intake of important nutritional components such as fibre and wholegrains regulate metabolic pathways associated with glycaemic control^2^; these may partly explain the modifying effect on genetic predisposition to high HbA1c and T2D risk. Proposed mechanisms from high‐fibre intake (wholegrains) may involve inhibition of a‐amylase, and slowdown in intestinal transit times, leading to a prolongation and reduction of intestinal glucose absorption and thus to a lower postprandial insulin secretory demand. Fibre may also stimulate gastrointestinal hormone secretion of gastric inhibitory polypeptide and glucagon‐like peptide‐1 (GLP‐1).^34^ Variants of *TCF7L2* lead to impaired GLP‐1‐induced insulin secretion via defects in GLP‐1 signalling in beta cells^35^ but individuals with a diet high in fibre (wholegrains) may counteract these genetic effects.

### 4.2 Strengths and limitations of study

The main strength of this study is the extensive dietary data collection (7‐day food diaries) and diet profiling on a large sample of white British population (of North European ancestry). A recent study from EPIC‐InterAct did not find interactions between a T2D‐associated GRS and intake of eight macronutrients, but food frequency questionnaires, based on predefined food lists, were used for dietary data collection rather than more detailed prospective food records. Furthermore, it did not include a holistic diet profile incorporating diet quality and patterns, which was used in our study.^14^ Another strength of this study was the use of a comprehensive GRS (based on established markers), which has the advantage over single markers as it increases the power of the genetic prediction model.^4^, ^8^ This is especially important for genetic interaction studies where effect sizes are very small. An important limitation is that our study design does not allow for determination of the temporality of the observed associations, which is important when considering the longitudinal dimension of T2D development and the complexity of nutrient and genetic interactions. Another limitation to this study is the use of perspective diet quality profiling in epidemiological study; however, given the lack of RCTs investigating differences in diet profile interactions with genetic risk, our study provides evidence to inform future intervention trials.

These findings need to be further evaluated and replicated in other settings applying the same method to confirm the interaction effects from diet and lifestyle on the genetic prediction of HbA1c. Finally, we did not apply stringent control for the limited number of multiple testing, and this may lead to potential overstatement of the findings. However, argument being that most of the nutritional variables are strongly correlated therefore not independent.

### 4.3 Implications of findings and conclusion

The findings suggest that healthy weight and a diet high in wholegrains may help counter the genetic risk of elevating HbA1c and support recommendations for healthy diet and weight management as part of public health prevention of T2D, especially in high‐genetic‐risk groups. Follow‐up studies and RCTs are needed to support these findings and to evaluate whether implementation of therapeutic dietary recommendations for people at high genetic risk may benefit from a “personalized nutrition” approach.

## CONFLICT OF INTEREST

None to declare.

## AUTHOR CONTRIBUTION

GF, RE, RG, QC and PE formulated the research question and methodological design; RE was responsible for data analysis and drafting of the manuscript. GF, PE, RG and QC contributed to the interpretation of results and final manuscript. RE and RG all contributed to the development of the food diary coding protocol, coder training and audit checking. QC assisted RE and RG with the dietary data extraction and cleaning. MA were responsible for the validation of the nondietary data extracts used in the analyses. EE and HG were responsible for the genotyping and imputation of the genetic dataset. PE is the principal investigator of the Airwave Health Monitoring Study. All authors read and approved the final manuscript.

## Data Availability

The data that support the findings of this study are available from ICHTB Tissue Management Committee. Restrictions apply to the availability of these data, which were used under licence for this study. Data are available [https://www.police-health.org.uk/research/data-access-enquiry] with the permission of ICHTB Tissue Management Committee.
