# Supplementary material for: Gene‐diet quality interactions on haemoglobin A1c and type 2 diabetes risk: The Airwave Health Monitoring Study
Source: Endocrinol Diabetes Metab. 2019 Jul 11;2(4):e00074. doi: 10.1002/edm2.74 (PMC6775444; doi:10.1002/edm2.74)
Supplement: Supplementary file 1 [file EDM2-2-e00074-s001.docx]

| **Supplementary material**  **Table S1: Socio-demographic and lifestyle characteristics across genetic risk score tertiles for men and women,**  **the Airwave Health Monitoring Study (n=14085)** | | | | | | | |
| --- | --- | --- | --- | --- | --- | --- | --- |
| **Genetic risk** | **Tertile 1^a^** | **Tertile 2^a^** | **Tertile 3^a^** | **P-value ^b^** | |  |  |
|  | Lowest risk |  | Highest risk |  | |  |  |
|  | *n 3434* | *n 3606* | *n 3563* |  | |  |  |
| Sex (female) | 39.77 | 39.32 | 38.16 | 0.3 | |  |  |
| Age (years) | 41.52 | 41.37 | 41.47 | 0.9 | |  |  |
| Diet quality score† | 4.66 | 4.61 | 4.52 | 0.5 | |  |  |
| Alcohol† |  |  |  |  | |  |  |
| No alcohol % | 20.02 | 21.23 | 21.22 | 0.7 | |  |  |
| Within UK allowance % | 44.56 | 44.77 | 44 |  | |  |  |
| Above UK allowance % | 35.42 | 34 | 34.78 |  | |  |  |
| Cigarette smoking |  |  |  |  | |  |  |
| Never smoker % | 67.95 | 68.18 | 67.15 | 0.2 | |  |  |
| Ex-smoker % | 24.47 | 24.17 | 23.72 |  | |  |  |
| Current smoker % | 7.58 | 7.65 | 9.13 |  | |  |  |
| Physical active (METs) |  |  |  | 0.9 | |  |  |
| Low (<600min/week) % | 9.8 | 10.16 | 8.98 | 0.3 | |  |  |
| Moderate (>600min/week) % | 40.2 | 39.02 | 39.39 |  | |  |  |
| High (>3000min/week) % | 50.01 | 50.82 | 51.63 |  | |  |  |
| Body Mass Index (kg/m2) | 26.92 | 27.05 | 27.02 | 0.6 | |  |  |
| Waist circumference (cm) | 88.96 | 89.17 | 89.27 | 0.7 | |  |  |
| Cardiovascular diseases and diabetes |  |  |  |  | |  |  |
| Type 2 Diabetes % | 3.19 | 3.69 | 4.44 | 0.03 | |  |  |
| Prediabetes % | 16.71 | 17.78 | 18.55 | 0.03 | |  |  |
| Hypertension % | 24.48 | 25.53 | 24.19 | 0.06 | |  |  |
| Dyslipidaemia % | 24.48 | 25.53 | 25.19 | 0.3 | |  |  |
| Abbreviations: METs, metabolic equivalent of task;  a. Values are unadjusted means or prevalence | | | | | | |  |
| b. P-value for differences between tertiles by linear regression (continuous variables) or Mantel-Haenzel chi-square test (categorical variables) | | | | | | | |
| * dietary dataset n=3733 |  |  |  |  |  | |  |

## Table S2: List of 87 single nucleotide polymorphisms included in the genetic risk score

| Genetic marker (rs-ID) | Mapped gene | Chromosome position | Risk allele associated with T2D/HbA1c | |
| --- | --- | --- | --- | --- |
| rs17791513 | GRB14 | 9:81905590 | A |  |
| rs7034200 | GIPR | 9:4289050 | A |  |
| rs10965250 | FN3KRP | 9:22133284 | G |  |
| rs3829109 | P2RX2 | 9:139256766 | G |  |
| rs16913693 | MADD | 9:111680359 | T |  |
| rs11782386 | NOTCH2 | 8:9201787 | C |  |
| rs2126259 | UBA52P6 - DMRTA1 | 8:9185146 | T |  |
| rs4841132 | HHEX - EXOC6 | 8:9183596 | A |  |
| rs983309 | CAMK1D | 8:9177732 | T |  |
| rs6474359 | ZFAND6 - FAH | 8:41549194 | T |  |
| rs13266634 | H1P1 | 8:118184783 | T |  |
| rs1167800 | ADCY5 | 7:75176196 | A |  |
| rs6943153 | PPP1R3B | 7:50791579 | T |  |
| rs730497 | SLC2A2 | 7:44223721 | A |  |
| rs849134 | ZMIZ1 | 7:28196222 | G |  |
| rs864745 | GCKR | 7:28180556 | C |  |
| rs1074708 | SLC30A8 | 7:159102498 | T |  |
| rs972283 | SLC30A8 | 7:130466854 | A |  |
| rs9502570 | MTNR1B | 6:7258617 | T |  |
| rs6912327 | YSK4 | 6:34764922 | T |  |
| rs3132524 | RPSAP52 | 6:31136714 | C |  |
| rs1800562 | RPL29P30 - LARP6 | 6:26093141 | G |  |
| rs4712523 | KCNQ1 | 6:20657564 | G |  |
| rs6937795 | IKBKAP | 6:137291281 | A |  |
| rs2745353 | HK1 | 6:127452935 | T |  |
| rs6235 | FAF1 | 5:95728898 | G |  |
| rs4457053 | MPHOSPH9 | 5:76424949 | G |  |
| rs702634 | FADSI | 5:53271420 | A |  |
| rs3822072 | KRT18P24 - CHCHD2P9 | 4:89741269 | A |  |
| rs4458523 | HFE | 4:6289986 | G |  |
| rs4691380 | PPARG | 4:157720124 | C |  |
| rs6813195 | PPP1R3B | 4:153520475 | C |  |
| rs9884482 | PDX1 | 4:106081636 | C |  |
| rs4607103 | KCNQ1 | 3:64711904 | C |  |
| rs7612463 | RNA5SP94 - MIR4432 | 3:23336450 | C |  |
| rs6808574 | GLS2 | 3:187740523 | C |  |
| rs4402960 | RSPO3 | 3:185511687 | T |  |
| rs11920090 | LYPLAL1 | 3:170717521 | T |  |
| rs1801282 | POU5F1 | 3:12393125 | C |  |
| rs11717195 | PROX1 | 3:123082398 | T |  |
| rs855791 | WARS | 22:37462936 | A |  |
| rs4812829 | FAM13A1 | 20:42989267 | A |  |
| rs6072275 | LOC728489 | 20:39743905 | A |  |
| rs6113722 | IGF2BP2 | 20:22557099 | G |  |
| rs243021 | HNF1B | 2:60584819 | A |  |
| rs7578597 | ZBED3-AS1 | 2:43732823 | T |  |
| rs1260326 | WFS1 | 2:27730940 | T |  |
| rs7578326 | NPM1P47 - C2CD4B | 2:227020653 | A |  |
| rs560887 | TCF7L2 | 2:169763148 | T |  |
| rs10195252 | ADAMTS9-AS2 | 2:165513091 | T |  |
| rs1530559 | PDGFC | 2:135755629 | A |  |
| rs10423928 | CDKAL1 | 19:46182304 | A |  |
| rs731839 | SGSM2 | 19:33899065 | G |  |
| rs12970134 | HNF4A | 18:57884750 | A |  |
| rs1046896 | PPP1R3B | 17:80685533 | T |  |
| rs4430796 | KCNJ11 | 17:36098040 | G |  |
| rs4790333 | G6PC2 | 17:2262703 | T |  |
| rs9936385 | KL | 16:53819169 | C |  |
| rs8042680 | TOP1 | 15:91521337 | C |  |
| rs11634397 | FOXA2 | 15:80432222 | G |  |
| rs7178572 | PCSK1 | 15:77747190 | G |  |
| rs1549318 | ANK1 | 15:71109147 | T |  |
| rs4502156 | BCL6 - LPP-AS2 | 15:62383155 | T |  |
| rs3783347 | RPS3AP18 - RPS14P6 | 14:100839261 | G |  |
| rs576674 | UHRF1BP1 | 13:33554302 | G |  |
| rs2293941 | SLC35D3 - RPL35AP3 | 13:28491198 | A |  |
| rs7998202 | GRB10 | 13:113331868 | G |  |
| rs1531343 | ARL15 | 12:66174894 | C |  |
| rs2657879 | GLIS3 | 12:56865338 | G |  |
| rs1727313 | HK1 | 12:123640853 | C |  |
| rs1387153 | HMG20A | 11:92673828 | T |  |
| rs174550 | GCK | 11:61571478 | T |  |
| rs10838687 | PEPD | 11:47312892 | T |  |
| rs163184 | LOC646736 | 11:2847069 | G |  |
| rs231362 | THADA | 11:2691471 | G |  |
| rs5215 | UBE2E2 | 11:17408630 | C |  |
| rs1111875 | RBMX2P4 - RPL26P21 | 10:94462882 | C |  |
| rs12571751 | ATP11AUN | 10:80942631 | A |  |
| rs7072268 | TSPAN8 - LGR5 | 10:71099913 | T |  |
| rs16926246 | LAMA1 | 10:71093392 | C |  |
| rs11257655 | TMPRSS6 | 10:12307894 | T |  |
| rs4506565 | JAZF1 | 10:114756041 | T |  |
| rs7901695 | TP53INP1 | 10:114754088 | C |  |
| rs17106184 | RREB1 - SSR1 | 1:50909985 | G |  |
| rs2785980 | PPP1R3B | 1:219700519 | T |  |
| rs340874 | TET2 | 1:214159256 | C |  |
| rs10923931 | KLF14 - MIR29A | 1:120517959 | T |  |

**Table S3: Genetic risk scores correlation with adiposity markers and HbA1c, the Airwave Health Monitoring Study (n 3733)**

|  | **GRS1 ^T2D+FTO^** | | **GRS2 ^T2D^** | |  |
| --- | --- | --- | --- | --- | --- |
|  | *r* | P value | *r* | P value |  |
| Body mass index | 0.03 | 0.001 | 0.01 | 0.3 |  |
| Waist circumference | 0.02 | 0.01 | 0.02 | 0.2 |  |
| Waist-to-hip | 0.03 | 0.04 | 0.03 | 0.1 |  |
| Body fat percentage | 0.02 | 0.4 | 0.01 | 0.6 |  |
| HbA1c | 0.06 | <.0001 | 0.06 | <.0001 |  |
| Abbreviation: GRS1, genetic risk score including single polymorphisms in FTO gene; GRS2, genetic risk score excluding single polymorphisms in FTO gene; HbA1c, glycated haemoglobin; *r*, Spearman correlation coefficient | | | | | |

**Table S4: The effect of genetic-diet quality interactions on HbA1c across genetic risk score (including FTO gene) tertiles, the Airwave Health Monitoring Study (n=3699)**

|  | GRS Tertile 1 | GRS Tertile 2 | GRS Tertile 1 |  |  |
| --- | --- | --- | --- | --- | --- |
|  | *Lowest risk* |  | *Highest risk* |  | |
|  | n 1263 | n 1116 | n 1320 |  | |

| Dietary components |  | |  | β ^a^ | 95% CI | β ^a^ | 95% CI | P *_interaction_* |  |
| --- | --- | --- | --- | --- | --- | --- | --- | --- | --- |
| DRV score † | | *ref* |  | -0.01 | -0.03, 0.001 | -0.01 | -0.02, 0.01 | 0.18 |  |
| Carbohydrates (per 10g) | | *ref* |  | -0.001 | -0.01, 0.005 | -0.04 | -0.01, 0.002 | 0.41 |  |
| Fibre (per 10g) | | *ref* |  | -0.04 | -0.1, 0.6 | -0.04 | -0.1, -0.004 | 0.71 |  |
| Fruit, vegetable (per 100g) | | *ref* |  | -0.01 | -0.03, -0.001 | -0.01 | -0.03, 0.01 | 0.4 |  |
| Wholegrains (per 100g) | | *ref* |  | -0.1 | -0.02, 0.01 | -0.03 | -0.1, 0.01 | 0.09 |  |
| Total fat (per 10g) | | *ref* |  | 0.001 | -0.01, 0.02 | 0.01 | -0.01, 0.02 | 0.9 |  |
| Saturated fat (per 10g) | | *ref* |  | 0.01 | -0.03, 0.05 | 0.002 | -0.04, 0.05 | 0.9 |  |
| Added sugars (per 10g) | | *ref* |  | -0.006 | -0.01, 0.01 | -0.006 | -0.02, 0.001 | 0.33 |  |

| Abbreviations: GRS, Genetic risk score; DRV, Dietary Reference Value score; β, beta-coefficient; CI, confident interval; P interaction; p-value type III error  ^a^ Estimated effect on HbA1c% per increase in nutrient variable interaction with GRS tertiles adjusted for age, gender, smoking, alcohol, energy intake, physical activity, BMI, diabetes diagnosis and treatment.  **†** coefficients represent per 1 point increase in DRV score |
| --- |

**Systematic literature review and selection of genetic markers**

A combined systematic literature review on published genome wide association studies (GWAS) was conducted using four search databases (a) PubMed, (b) DIAbetes Genetics Replication And Meta-analysis consortium (DIAGRAM), (c) Meta-Analyses of Glucose and Insulin-related traits Consortium (MAGIC) and (d) National Human Genome Institute Research (NHGIR) GWAS database. The review aimed to find published GWAS on common SNPs associated phenotype T2D and glycaemic trait HbA1c.

Each database was searched systematically using search key words defined in figure 1. The output of the search produced 180 papers, which were reviewed based on a set of selected inclusion/exclusion criteria (figure 2) and reduced to 37 (20 duplicated) papers. From the reviewed papers a list of 161 SNPs represented different loci found to have a genome-wide statistical significance level (p<5x10^-8^) associated with hyperglycaemia trait HbA1c or/and phenotype T2D. The generated list of 161 SNPs included mapped genes, risk alleles, chromosome position, coding region, original cohort effect size and p-values and was inputted into Microsoft Excel for further data cleaning.

**Figure 1: Search key words for systematic literature review**

| **Phenotype** | **Phenotypic trait** | **Genome wide association studies** |
| --- | --- | --- |
| “T2D” OR “type 2 diabetes” | “HbA1c” OR “glycated haemoglobin A1c” | “genome-wide" OR "genome AND identification" OR "genome AND association” |

Abbreviations: T2D, type 2 diabetes; HbA1c, glycated haemoglobin A1c

**Figure 2: Search criteria systematic literature review**

| Inclusion criteria | Papers published in English  Original research articles  Human studies: Caucasian population (European ancestry)  GWAS papers including replicated studies and/or meta-analysis  Additional SNP criteria  Known and common disease-associated SNPs of functional significance to phenotype T2D and glycaemic trait HbA1c  SNPs with a genome-wide statistical significance level (p<5x10^-8^)  SNPs located inside a known gene: regulatory or coding region |
| --- | --- |
| Exclusion criteria | Publication dates > 5 years  Non-European population groups or not stated  Diabetes type 1 and gestational diabetes  Novel and rare SNPs  SNPs located in known obesity genes |

Abbreviations: SNP, single nucleotide polymorphism

## Systematic literature review data management and cleaning

The list was further quality checked to avoid any overlapping with obesity SNPs and LD pairwise with other SNPs coding regions (e.g. coding for the same outcome), which may cause over emphasising SNPs effect. This was done to ensure only independent SNPs and SNPs with the highest LD, strongest association with phenotype remained.

SNPs’ coding regions were checked using UCSC Genome Browser on Human version GRCh38/hg38 (date Dec. 2013)(1). SNPs with overlapping regions were highlighted and their pairwise LD was calculated. Pairwise LD calculations were done in an online genetic variant-centered annotation database browser, SNIPA version 3.1 (released November 2015)(2). SNIPA estimates how often the SNPs appear in pairwise LD (distant limit set at 500 kb) tested against a reference population panel from the 1,000 Genomes (pilot), 1,000 Genome Project, Phase 3 v 5 Linkage disequilibrium data (sample of Utah residents with ancestry from northern and western Europe). *r*^2^ threshold between the SNPs was determined as high correlation if *r*^2^ > 0.8 and D-prime threshold was set at >0.8, which determine if SNPs LD are co-inherited >80% of the time (3, 4). Those with the lowest *r*^2^ value were excluded to ensure the SNPs with the strongest reported correlation with phenotype were used. Further LD pruning was conducted to SNPs on the same chromosome (not in same coding regions) to determine if they were in close LD with each other. The check excluded 64 SNPs. Additional 6 SNPs (rs9939609, rs1421085, rs8090011, rs8050136, rs11642841, rs12970134) were positioned within obesity-associated genes FTO and MC4R and were therefore excluded given the well-documented primary association with BMI, which mediates the effect on T2D risk (3, 5, 6).

**Protocol for linkage disequilibrium pruning of genetic markers**

1. Removed SNPs within the same coding region. Checked SNPs coding region on database UCSC Genome Browser
2. Removed SNPs in close LD. Calculated LD using database SNIPA

Input SNP’s rs-ID (text entry) and set the database search criteria:

- 1. Distance limit 500 kb
  2. Population panel: CEU (European ancestry)
  3. Correlation between the SNPs *r*^2^ threshold > 0.8 and D-prime > 0.8
  4. 1000 Genomes (pilot) variant dataset was used for computation of allele frequencies for the selected variant set. This was double-checked with other variant datasets 1,000 Genome Project and Phase 3 v 5 Linkage disequilibrium.

The remaining 91 SNPs were checked against the SNPs in the Airwave Health Monitoring study’s genetic dataset of which the majority were found genotyped, with exception of 1 SNPs rs11603334 not genotyped. A further 3 SNPs were excluded due to chromosome position: rs5945326 positioned in x chromosome X:152899922 and no SNPs corresponded with 3:49455330, 1:158585415.

The final list of 87 SNPs consisted of 44% (n=38) SNPs associated with HbA1c or/and other glycaemic traits and 56% (n=49) T2D associated SNPs.

##

## Genetic data quality control

The genetic data is cleaned and analysed in PLINK based on published protocol (4, 7) and based on previous published papers quality control (4, 8).

**Minor Allele Frequencies (MAF)**

SNPs were tested for MAF. MAF is the frequency at which the minor allele (or less abundant) of a SNP is present in a population based on comparisons of estimated frequencies from a sample population e.g. The International HapMap Project (applied in PLINK). The MAF for a SNP to be considered common is usually >1% (3, 9). All SNPs passed the MAF frequency test p<0.01.

**Hardy-Weinberg equilibrium**

All SNPs were tested for the Hardy-Weinberg equilibrium, which detects if a genotype can be due to a sequencing error or batch effect. It tests the frequency of different alleles and genotypes in a population, which are in genetic equilibrium. Genetic equilibrium is defined as remain constant from generation to generation in the absence of evolutionary influence e.g. mutations. The null hypothesis test if the sample is the same as expected i.e. the probability of the population being in Hardy-Weinberg equilibrium at that genotype. SNPs > 0.001 will be rejected and removed from the dataset (4). 1 SNP failed the Hardy-Weinberg equilibrium test and was excluded from this study.

**Missingness test**

Missingness rates per SNP were checked to exclude SNPs with more than 1% missing genotype data. None of the selected SNPs failed the missingness test criteria threshold <0.01 (4).

Missingness rates per person were also checked to only include participants with over 99% of the tested SNPs. The total genotyping rate was 0.98 resulting in 282 participants being removed due to failing the criteria p<0.01 (4).

**Other quality control tests**

Population stratification (controlling for confounding by ethnicity and ancestry) was not applicable in this quality control as the dataset only consists of white British population of European ancestry. Hence, assumptions were made that minimum variation or genetically distinct differences existed within subgroups of this study population (10). Furthermore, the SNPs applied were extracted from replicated GWAS datasets where population stratification was applied in their quality control.

Heterozygosity (controlling for proportion of heterozygosity within the SNPs) was not applicable either in this dataset as it was assumed that the structure of the sample population consisted of individuals from different families, i.e. no first-degree relatives*.* The individuals in the Airwave Health Monitoring study population were expected to have a low proportion of heterozygosity within the markers (no outlier > 3SD from the mean). Heterozygosity is sensitive to population structure because allele frequency distribution (*heterozygosity)* can differ between different populations (4, 7).

In summary, the quality control of the genetic data excluded 284 participants based on genotyping rate < 98% and 1 SNP was removed due to failed the Hardy-Weinberg equilibrium test > 0.001. The remaining sample consisted of 14,089 individuals with clinical data of which 3,733 also had dietary data. The final list of 87 SNPs, which passed the quality checked was exported and prepared for GRS calculations.

**References**

1. Kent WJ, Sugnet CW, Furey TS, Roskin KM, Pringle TH, Zahler AM, et al. The human genome browser at UCSC. Genome research. 2002;12(6):996-1006.

2. Arnold M, Raffler J, Pfeufer A, Suhre K, Kastenmüller G. SNiPA: an interactive, genetic variant-centered annotation browser. Bioinformatics. 2014:btu779.

3. Morris AP, Voight BF, Teslovich TM, Ferreira T, Segre AV, Steinthorsdottir V, et al. Large-scale association analysis provides insights into the genetic architecture and pathophysiology of type 2 diabetes. Nature genetics. 2012;44(9):981.

4. Genotyping and quality control of UK Biobank, a large-scale, extensively phenotyped prospective resource. Oct 2015. Available from: <http://www.ukbiobank.ac.uk/wp-content/uploads/2014/04/UKBiobank_genotyping_QC_documentation-web.pdf>.

5. Fall T, Hägg S, Mägi R, Ploner A, Fischer K, Horikoshi M, et al. European Network for Genetic and Genomic Epidemiology (ENGAGE) consortium. The role of adiposity in cardiometabolic traits: a Mendelian randomization analysis. PLoS Med. 2013;10(6):e1001474.

6. Karaderi T, Drong AW, Lindgren CM. Insights into the Genetic Susceptibility to Type 2 Diabetes from Genome-Wide Association Studies of Obesity-Related Traits. Curr Diab Rep. 2015;15.

7. Anderson CA, Pettersson FH, Clarke GM, Cardon LR, Morris AP, Zondervan KT. Data quality control in genetic case-control association studies. Nature protocols. 2010;5(9):1564-73.

8. Eyheramendy S, Juhanson P, Gieger C, Lichtner P, Klopp N, Veldre G, et al. Genome-wide scan identifies CDH13 as a novel susceptibility locus contributing to blood pressure determination in two European populations. Human molecular genetics. 2009;18(12):2288-96.

9. Gibbs RA, Belmont JW, Hardenbol P, Willis TD, Yu F, Yang H, et al. The international HapMap project. Nature. 2003;426(6968):789-96.

10. Purcell S NB, Todd-Brown K, Thomas L, Ferreira MAR, Bender D, Maller J, Sklar P, de Bakker PIW, Daly MJ & Sham PC , . PLINK 1.07: a toolset for whole-genome association and population-based linkage analysis. American Journal of Human Genetics. 2007 81.
